# Role of Tim-3 in Decidual Macrophage Functional Polarization During Abnormal Pregnancy With *Toxoplasma gondii* Infection

**DOI:** 10.3389/fimmu.2019.01550

**Published:** 2019-07-11

**Authors:** Dan Zhang, Liqin Ren, Mingdong Zhao, Chunyan Yang, Xianbing Liu, Haixia Zhang, Yuzhu Jiang, Xinyue Sun, Teng Li, Lijun Cui, Xuemei Hu

**Affiliations:** ^1^Department of Immunology, Binzhou Medical University, Yantai, China; ^2^Medicine and Pharmacy Research Center, Binzhou Medical University, Yantai, China; ^3^Department of Radiology, Binzhou Medical University, Yantai, China

**Keywords:** Tim-3, decidual macrophage, polarization, *Toxoplasma gondii*, abnormal pregnancy outcomes

## Abstract

Vertical transmission of the intracellular parasite *Toxoplasma gondii* (*T. gondii*) can lead to devastating consequences during gestation. Tim-3, a negative immune regulator, is constitutively expressed on decidual macrophages, but its specific role during *T. gondii* infection has not yet been explored. In the present study, we discovered that Tim-3 plays an important role in the abnormal pregnancy due to *T. gondii* infection using Tim-3^−/−^ pregnant mice and anti-Tim-3 neutralizing antibody treated human decidual macrophages. The results showed that abnormal pregnancy outcomes were more prevalent in Tim-3^−/−^ infected pregnant mice than in wild-type infected pregnant mice. Tim-3 expression in decidual macrophages was significantly down-regulated after *T. gondii* infection both *in vitro* and *in vivo*. Tim-3 down-regulation by *T.gondii* infection could strengthen M1 activation and weaken M2 tolerance by changing the M1 and M2 membrane molecule expression, arginine metabolic enzymes synthesis, and cytokine secretion profiles of decidual macrophages. Moreover, Tim-3 down-regulation by *T.gondii* infection led to PI3K-AKT phosphorylation inhibition, downstream transcription factor C/EBPβ expression, and SOCS1 activation, which resulted in enzymes synthesis regulation and cytokines secretion. Our study demonstrates that Tim-3 plays an indispensable role in the adverse pregnancy outcomes caused by *T. gondii* infection.

## Introduction

Toxoplasmosis, a common parasitic zoonosis occurring worldwide, is caused by the obligate intracellular protozoan parasite, *Toxoplasma gondii* (*T.gondii*) (1). In addition to its common transmission route through accidental ingestion of water or food contaminated with oocysts, *T. gondii* can also be transmitted to humans congenitally during pregnancy. *T. gondii* infection during pregnancy can result in fetal and neonatal death or various congenital defects, especially when the congenital infection occurs acutely during the first trimester (2). However, the mechanism through which *T. gondii* infection causes these severe abnormalities during human pregnancy is not clear.

A successful pregnancy requires the maternal immune system to tolerate the semi-allogeneic fetus (3). A balanced immune response mediated by decidual immune cells is critical in this dynamic and highly regulated immunologic process (4). Within the maternal-fetal immune system, decidual macrophages, the second largest population of decidual leukocytes during the first trimester (~20%) after decidual NK cells (~70%), are required for a successful pregnancy, including trophoblast invasion, tissue and vascular remodeling, and permitting maternal-fetal tolerance throughout gestation (5–7). Macrophages are classified into type 1 (M1) and type 2 (M2) macrophages (8). During normal pregnancy, decidual macrophages have an immunosuppressive, M2-like phenotype, characterized by typical M2-associated markers (e.g., CD163, CD206, CD209, IL-10, and Arg-I). M2 cells are immunosuppressive and promote immune tolerance at the maternal-fetal interface (5, 9). The polarization pattern of decidual macrophages skews toward M1 during pregnancy-related disorders such as miscarriages or preeclampsia, as evidenced by their high expression of CD80, CD86, and TNF-α (10, 11). Our previous study has showed that *T. gondii* infection causes a bias toward M1 decidual macrophages, thus contributing to abnormal pregnancy (12). Moreover, the down-regulation of the inhibitory receptor, LILRB4, can strengthen M1 decidual macrophages activation, and consequently, cause severe adverse pregnancy outcomes during *T. gondii* infection (13). Whether there is any other immune molecule on macrophage participating in the process and contributing to the adverse pregnancy outcomes during *T. gondii* infection still needs further exploration.

T cell immunoglobulin domain and mucin domain (Tim)-3 was first described as a molecule specifically expressed on the surface of IFN-γ-producing Th1 and cytotoxic Tc1 cells (14). Tim-3 on T cells transduce apoptotic signals and are involved in tolerance induction (15). Recent studies have reported that Tim-3 is a key mediator in maintaining maternal-fetal immune tolerance and successful pregnancy (16, 17). As a negative immune regulator at the maternal-fetal interface, Tim-3 is constitutively expressed on decidual macrophages, blockage of Tim-3 results in the accumulation of macrophages and up-regulation of pro-inflammatory cytokines such as TNF-α, which elicits local inflammation (18). But whether *T. gondii* infection regulates Tim-3 expression level on decidual macrophages and contributes to adverse pregnancy outcomes still need to be confirmed.

The signaling pathways involved in macrophage immune regulation in response to Tim-3 dysregulation have been studied extensively. Tim-3 mediates the negative regulation of the innate immune response by inhibiting LPS-TLR4-mediated NF-κB activation; this occurs through an increase in PI3K-AKT phosphorylation, which finally upregulates TNF-α and IL-10 simultaneously (19). Tim-3 also promotes tumor-promoting M2 macrophage polarization by binding to STAT1, and it inhibits SOCS1, leading to a subsequent increase in IL-10 and Arg-I expression (20). However, little is known about the signaling pathway through which Tim-3 regulates the function of macrophages and contributes to adverse pregnancy outcomes due to *T. gondii* infection. In the present study, *T. gondii* infected human decidual macrophages and *T. gondii* infected Tim-3^−/−^ pregnancy mouse model were used to examine the role of Tim-3 and its immune mechanism in the adverse pregnancy outcomes caused by *T. gondii* infection.

## Materials and Methods

### Animal Models

Wild type (WT) mice (Beijing Vital River Laboratory Animal Technology, Co., Ltd.) and Tim-3^−/−^ mice (Bioray Laboratories Inc.) were maintained in our animal facility according to Institutional and National Institutes of Health guidelines. Six- to eight-week-old females were mated to eight- to ten-week-old males to induce pregnancy and inspected every morning for vaginal plugs. The day of visualization of a plug was designated as day 0 [gestational day (Gd) 0] of pregnancy. The infected group was inoculated intraperitoneally (i.p.) with 400 tachyzoites in 200 ml sterile PBS on Gd 8. The uninfected groups were inoculated with the same value of PBS at the same time. All procedures performed on animals in this study were conducted following the ethical standards formulated by the Ethics Committee and Institutional Animal Experimental Ethics Committee of Binzhou Medical University.

### Scanning Electron Microscopy (SEM)

The mice were sacrificed on Gd 14, the fetuses were removed and washed 5–6 times in phosphate buffer (0.1 M); then, the fetuses were immobilized with 2.5% phosphate buffered glutaraldehyde at 4°Cfor 2 days. Immobilized fetuses were placed on polylysine-coated glass coverslips and dehydrated using a graded ethanol series, being immersed for 10 min at each step. The samples were dried by the critical point technique (Quorum K850), attached to specimen holders and coated with gold particles using an ion sputter coater (Quorum Q150RS). The specimens were observed with a scanning electron microscope (ZEISS EVO LS15) operated at 10 KV. All images were obtained using the SmarSEM user interface software.

### Genotyping

Genomic DNA was extracted from mouse tails using a tissue DNA extraction kits (Generay, China). The polymerase chain reaction (PCR) was used to synthesize cDNA. After an initial denaturation (3 min at 95°C), PCR was performed with 35 amplification cycles of denaturation for 30 s at 95°C, annealing for 30 s at 55°C, and extension for 60 s at 72°C, followed by a final extension for 5 min at 56°C. Primers for PCR amplification were Tim-3 F, 5′-GGCTGGCTCAAACTCACTACA-3′ and Tim-3 R, 5′-CGGACAATGATAACATGGAAA-3′. After sequencing the PCR products (Shanghai Majorbio Bio-Pharm Technology Co., Ltd), we distinguish the homozygote from heterozygote or WT mice by analyzing the DNA chromatogram. Homozygous Tim-3^−/−^ mice were continually bred for the duration of the study.

### Maintenance of *T. gondii* Tachyzoites (RH Strain)

The *T. gondii* tachyzoites were cultured in HEp-2 cells growing in minimum essential media (MEM) (Hyclone, United States), 5% fetal bovine serum (FBS; Gibco, United States), and 100 IU/ml penicillin/streptomycin (Sigma-Aldrich, United States). Cultured tachyzoites were centrifuged at 1,500 rpm for 10 min, and purified tachyzoites were resuspended in MEM and counted using a Neubauer chamber. The experiment was carried out in BSL-2 laboratories. All the liquids, consumables, and labwares contaminated with the parasites were collected, steeped immediately in disinfectant, and autoclaved.

### Cell Preparation

Uteri and placenta from pregnant mice were separated from the mesometrium and dissected with scissors to remove fetuses, which were washed twice in ice-cold PBS and shredded carefully by using a GentleMACS dissociator (Miltenyi, Germany). Single cell suspensions were obtained by filtration through 48 μm sterile nets, and mononuclear cells was obtained via density gradient centrifugation. The collected cells were washed with PBS and immediately used for flow cytometry analyses. The mouse carcasses were collected in an ice chest and transported out by professional public health workers.

### Human Clinical Sample Collection

Decidual tissues were collected from 30 voluntary abortion cases in the first trimester [gestational age at 8–10weeks], after informed consent were given. The sample collection for this study was approved by the Ethics Committee of Binzhou Medical University. All subjects were visiting the Department of Obstetrics and Gynecology, Yantai Affiliated Hospital of Binzhou Medical University, had not used any abortifacient or suffered from any pregnancy complication. The tissues were immediately washed with sterile saline solution 5–8 times and decidual tissues were carefully separated from villi under sterile conditions. These were then cultured in DMEM/high glucose medium (Hyclone, USA) which containing 10% fetal bovine serum (FBS,Gibco, USA), 100 IU/ml penicillin, and 100 IU/ml streptomycin (Sigma-Aldrich, USA).

### Isolation and Purification of Human Decidual Macrophages

Pieces of decidual tissue were minced using the Gentle MACS tissue dissociator (Miltenyi Biotec, Germany) according to the manufacturer's instructions, and the resulting suspension was filtered through 48-um nylon mesh filters. The mononuclear cells were subsequently purified on a Ficoll-Hypaque gradient (SigmaAldrich, United States) at 2,000 rpm for 20 min at 20°C. To purify CD14^+^ decidual macrophages, the mononuclear cells were subjected to immunomagnetic positive selection (Stem Cell Science, USA), resulting in purity levels of more than 95%. Approximately 1.5 × 10^6^ purified decidual macrophages were allocated to uninfected, infected, and Tim-3-neutralized infected groups. CD14^+^ cells were incubated with 10 μg/mL of an anti-Tim-3 monoclonal antibody (mAb) (eBioscience, USA) in the Tim-3-neutralized infected group. One hour later, *T. gondii* tachyzoites were added to the Tim-3-neutralized infected group and the infected group at a ratio of 2:1 (*T. gondii*: cells). Study samples were cultured in RPMI medium supplemented with 10% FBS (FBS; Gibco, USA), 100 IU/ml streptomycin, and 100 IU/ml streptomycin (Sigma, USA) for 24 h at 37°C in a humidified 5% CO_2_ incubator.

### Flow Cytometry

The following mouse-specifc mAbs were used: Pe-cy7-conjugated anti-F4/80, PE-conjugated anti-CD206, APC-conjugated anti-TNF-α (all from Biolegend, USA), APC-conjugated anti-Tim-3, FITC-conjugated anti-CD80, APC-conjugated anti-iNOS (all from eBioscience, USA), PE-conjugated anti-CD86, APC-conjugated anti-IL-10 (all from BD, USA), and APC-conjugated anti-Arg-I (RD, USA).

The following human-specific (mAbs) were used: Pe-cy7-conjugated anti-CD14, APC-conjugated anti-Tim-3, and PE-conjugated anti-TNF-α were purchased from eBioscience; FITC-conjugated anti-CD206, FITC-conjugated anti-CD163, FITC-conjugated anti-CD209, PE-conjugated anti-CD80, PE-conjugated anti-CD86, and PE-conjugated anti-IL-10 were purchased from BD company.

The mice decidual lymphocytes or human decidual macrophages were incubated with corresponding mAbs at 4°C in the dark for 30 min and were then washed once; intracellular cytokine and enzyme staining were performed after cellular fixation and permeabilization as previously described (13). Analysis was performed with a FACScanto^TM^ II instrument (Becton Dickinson, USA).

### Western Blot Analysis

Equal amounts of protein from total-cell lysates were separated by 12% SDS-PAGE (Beyotime) and transferred onto polyvinylidene difluoride membranes. The membranes were blocked at room temperature (20–25°C) for 2.5 h in 5% non-fat dry milk in TBS-T. Membranes were incubated with gentle rocking 1.5 h at room temperature with primary antibodies for Tim-3 (1:2,000, Proteintech, China), Arg-I (1:1,000, Proteintech, China), iNOS (1:1,000, Abcam, UK), PTEN (1:600, Proteintech, China), PI3K (1:500, Proteintech, China), AKT (1:500, SAB, USA), pAKT (1:500, SAB, USA), SOCS1 (1:600, Proteintech, China), C/EBPβ (1:750, SAB, USA), TNF-α (1:2,000, Proteintech, China), IL-10 (1:5,000, Proteintech, China); GAPDH (1:40,000, Proteintech, China) was used as a loading control. Membranes were washed 5 times with TBS-T for 10 min each time and then incubated with the appropriate secondary Ab for 2.5 h at room temperature. The immune complex was visualized with an enhanced chemiluminescence (ECL) detection kit (F. Hoffmann-La Roche, Ltd., Switzerland). Protein expression levels were determined using the Image J software (Rawak Software, Inc., Germany).

### Enzyme-Linked Immunosorbent Assays (ELISA)

The supernatants of purified human decidual macrophages from uninfected, infected, and Tim-3-neutralized infected groups were obtained and tested for TNF-α and IL-10 levels by ELISA according to the manufacturer's protocols (Enzyme-linked Biotechnology, China). Standard curves were generated using standards for each assay, and all measurements of absorbance were performed in triplicate at 450 nm. Concentrations were calculated according to standard curves and respective formulas.

### Statistical Analysis

Data are presented as the mean ± SEM. Statistical analysis was performed using the SPSS statistics software package (SPSS 17.0; SPSS, Inc., Chicago, IL, United States). Unpaired *t*-tests were used to compare the means of two independent groups after verifying that the data of the groups had a normal distribution using SAS. *p* < 0.05 was regarded as significant and *p* < 0.01 was considered as very significant.

## Results

### Abnormal Pregnancy Outcomes Were More Prevalent in *T. gondii-*Infected Tim-3^−/−^ Pregnant Mice Than in the Infected Wild-Type (WT) Pregnant Mice

**Figure 1:**
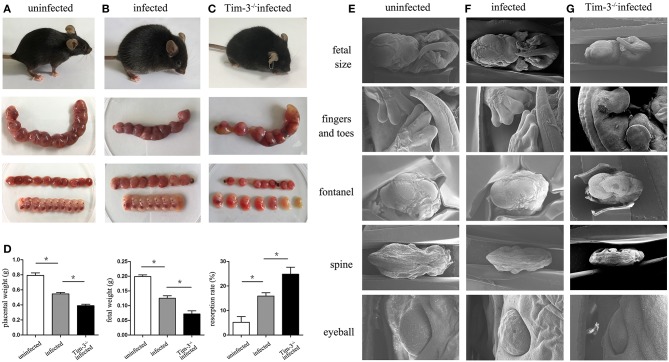
The impact of Tim-3 on abnormal pregnancy outcomes caused by *Toxoplasma gondii* infection in mice. **(A)** Uninfected mice were healthy and normal fetuses and placentas. **(B)** Infected mice were lethargic, and fetuses and placentas were inflamed with hyperemia. **(C)** Tim-3^−/−^ infected mice were spiritual malaise, and showed more absorbed fetuses. **(D)** The weight of placenta, fetus, and resorption rate were analyzed in uninfected, infected, and Tim-3^−/−^ infected mice. **(E,F,G)** The differences of fetal development among three groups were observed by scanning electron microscopy (SEM), including the development of fetal size, fingers and toes, fontanelles, spine, and eyeball. Data are presented as means ± SD of 8 pregnant mice per group. Asterisks indicate significant differences for unpaired *t*-tests; ^*^p < 0.05.

The pregnancy outcomes between Tim-3^−/−^ infected pregnant mice and *T. gondii*-infected WT mice were compared. The Tim-3^−/−^ infected pregnant mice were unkempt, had less mobility, erect fur, and placentas that were significantly inflamed with hyperemia. Tim-3^−/−^ infected mice were more susceptible to fetal loss as manifested by a decrease in placental and fetal weights and an increased rate of resorption compared with infected wild-type mice (Figure 1D). The placental ischemia, hyperemia, and fetal malformations were more severe in infected WT mice compared with uninfected mice (Figures 1A–C).

The differences of fetal development among the three groups were observed by scanning electron microscopy (SEM). Fetuses from the Tim-3^−/−^ infected mice were even smaller compared to those from infected pregnant mice (Figures 1E–G), which agrees with the previously observed fetal weight results of pregnancy outcomes. In addition, there were more severe dysplasia and hypoplasia in Tim-3^−/−^ infected mice, indicated by the unformed fingers and toes, early closed or bulged fontanelles, curved spine, and abnormal eyeball development.

### Tim-3 Expression on Decidual Macrophages Decreased After *T. gondii* Infection

**Figure 2:**
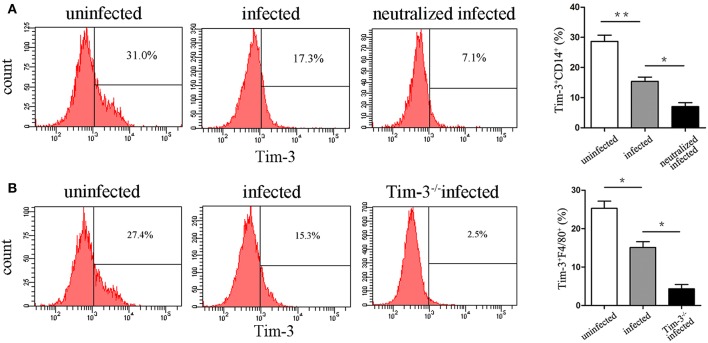
Expression changes of Tim-3 on decidual macrophages. **(A)** Flow cytometry analyses of Tim-3 dynamic expression on CD14^+^ human decidual macrophages were performed in uninfected, infected, and neutralized infected groups. **(B)** Flow cytometry analyses of Tim-3 dynamic expression on F4/80^+^ mouse decidual macrophages were performed in uninfected, infected, and Tim-3^−/−^ infected groups. Data are presented as means ± SD for each group, and differences were identified by unpaired *t*-test; *p < 0.05, **p < 0.01.

Flow cytometry was performed in purified human decidual macrophages or murine decidual lymphocytes after *T. gondii* infection. The results show that Tim-3 expression levels on decidual macrophages were both significantly decreased both *in vitro* (Figure 2A) and *in vivo* (Figure 2B) after *T. gondii* infection.

### Down-Regulation of Tim-3 by *T.gondii* Infection Is Associated With Changes of M1 and M2 Membrane Molecules of Decidual

**Figure 3:**
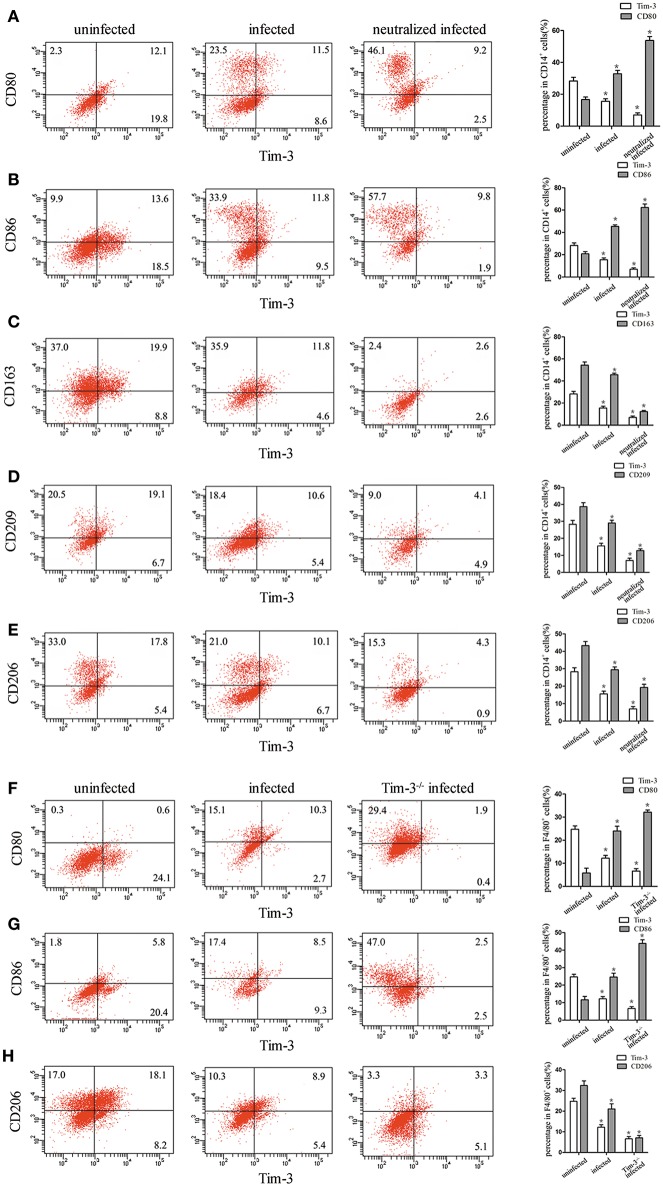
Downregulation of Tim-3 on human decidual macrophages infected with *Toxoplasma gondii* resulted in changes in the expression of M1 and M2 membrane functional molecules. Flow cytometry and histogram analyses of CD80 **(A)**, CD86 **(B)**, CD163 **(C)**, CD209 **(D)**, CD206 **(E)**, and Tim-3 expression of human decidual macrophages were tested in uninfected, infected, and neutralized infected groups. Flow cytometry and histogram analyses of CD80 **(F)**, CD86 **(G)**, CD206 **(H)**, and Tim-3 expression of mouse decidual macrophages were observed in uninfected, infected, and Tim-3^−/−^ infected groups. Data are presented as means ± SD for each group, and differences were identified by unpaired *t*-test; ^*^p < 0.05.

Tim-3 expression on human decidual macrophages was significantly lower in the *T. gondii* infected group than in the uninfected group. Also, in the *T. gondii* infected group, M1 membrane-functional molecules CD80 and CD86 were up-regulated (Figures 3A,B), while M2 membrane-functional molecules CD163, CD209, and CD206 were significantly down-regulated compared to the uninfected group. In order to explore whether the down-regulation of Tim-3 by *T. gondii* infection was associated with changes in M1 and M2 membrane molecules, an anti-Tim-3 neutralized antibody was added to infected human decidual macrophages. In the Tim-3 neutralized infected group, M1 membrane functional molecules were further up-regulated, whereas M2 membrane functional molecules were further down-regulated as compared to the infected cells (Figures 3C–E).

Similar to the results of *in vitro* studies, M1 membrane-functional molecules CD80 and CD86 were both significantly increased after *T. gondii* infection; and their levels increased further in Tim-3^−/−^ infected mice (Figures 3F,G). On the other hand, the M2 membrane-functional molecule CD206 was significantly decreased after *T. gondii* infection and its levels decreased further in Tim-3^−/−^ infected mice (Figure 3H).

### Tim-3 Down-Regulation by *T. gondii* Infection Is Involved in the Expression of Arginine Catabolism Enzymes iNOS and Arg-I of Decidual Macrophages

**Figure 4:**
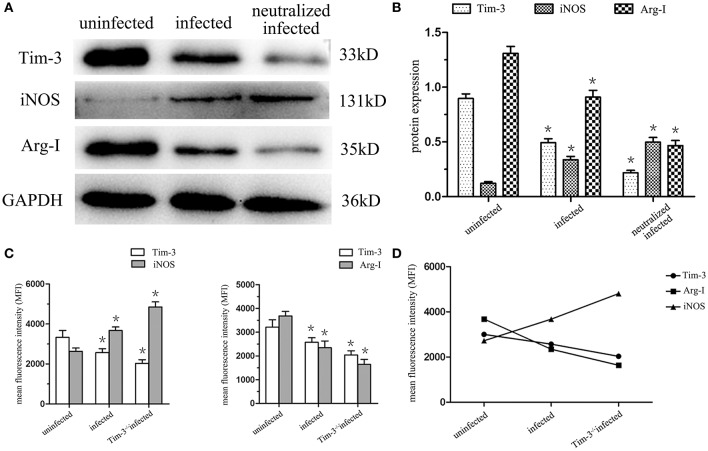
Downregulation of Tim-3 on decidual macrophages by *Toxoplasma gondii* infection changed the expression of the arginine catabolism enzymes type-I arginase (Arg-I) and inducible nitric oxide synthase (iNOS). **(A)** Representative analysis of iNOS and Arg-I protein levels in uninfected, infected, and Tim-3-neutralized infected human decidual macrophages by western blot. **(B)** Histograms analysis of western blot for iNOS and Arg-I expression in uninfected, infected, and Tim-3-neutralized infected human decidual macrophages. **(C)** Changes in the expression of iNOS and Arg-I followed Tim-3 on mouse decidual macrophages were compared in uninfected, infected, and Tim-3^−/−^ infected groups. **(D)** Relativity analysis of Tim-3, iNOS, and Arg-I expression in uninfected, infected and Tim-3^−/−^ infected groups. Data are shown as means ± SD of 8 samples for each group, and differences were identified by unpaired *t*-test; ^*^p < 0.05.

The expression of iNOS and Arg-I in human decidual macrophages were analyzed using western blotting, and the results showed that the arginine catabolism enzyme iNOS was almost undetectable in uninfected human decidual macrophages. After *T. gondii* infection, the enzyme level increased, and the increase was more significant in Tim-3-neutralizing antibody treated group. Arg-I synthesis was significantly reduced following *T. gondii* infection and was further reduced in the infected Tim-3-neutralized macrophages (Figures 4A,B). Under *in vivo* conditions, flow cytometric analyses of iNOS and Arg-I also measured similar expression changes as those seen *in vitro*. iNOS expression was significantly increased in decidual macrophages of *T. gondii* infected mice compared with uninfected mice, and a higher expression level was observed in decidual macrophages of Tim-3^−/−^ infected mice compared to WT infected mice. The expression of Arg-I was decreased in decidual macrophages of infected mice compared with uninfected mice, and further decreased in decidual macrophages of Tim-3^−/−^ infected mice compared with WT infected mice (Figures 4C,D).

### Effects of Decreased Tim-3 by *T. gondii* Infection on TNF-α and IL-10 Secretion of Decidual Macrophages

**Figure 5:**
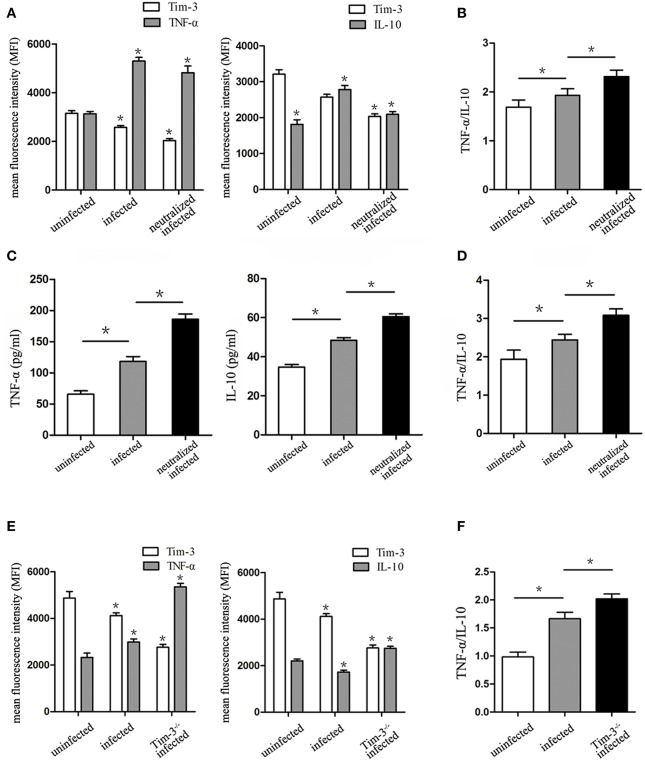
Downregulation of Tim-3 on decidual macrophages by *Toxoplasma gondii* infection caused changes of tumor necrosis factor alpha (TNF-α) and interleukin 10 (IL-10) synthesis and secretion by decidual macrophages. **(A)** Intracellular levels of TNF-α and IL-10 were assessed in uninfected, infected, and Tim-3-neutralized infected human decidual macrophages by flow cytometry analyses. **(B)** Ratios of TNF-α/IL-10 in uninfected, infected, and Tim-3-neutralized infected human decidual macrophages. TNF-α levels, IL-10 levels **(C)**, and TNF-α/IL-10 ratios **(D)** in supernatants were analyzed in uninfected, infected, and Tim-3-neutralized infected human decidual macrophages by enzyme-linked immunosorbent assays (ELISA). TNF-α levels, IL-10 levels **(E)**, and TNF-α/IL-10 ratios **(F)** of mouse decidual macrophages were observed in uninfected, infected, and Tim-3^−/−^ infected groups. Data are presented as means ± SD of 8 samples for each group, and differences were identified by unpaired *t*-test; ^*^p < 0.05.

The levels of M1 macrophage-associated cytokine (TNF-α) and M2 macrophage-associated cytokine (IL-10) were analyzed by flow cytometry and ELISA. The *in vitro* flow cytometry results show that as the expression of Tim-3 lowers, the TNF-α levels significantly elevate following *T. gondii* infection; however, these levels decrease in infected Tim-3-neutralized cells. Similarly, IL-10 levels within human decidual macrophages were also increased by *T. gondii* infection and reduced in infected Tim-3-neutralized cells (Figure 5A). TNF-α/IL-10 ratios were higher in infected cells than in uninfected cells and were further increased in infected Tim-3-neutralized cells (Figure 5B). ELISA results show an increased TNF-α secretion in supernatants from infected human decidual macrophages, which was further increased in supernatants from infected Tim-3-neutralized cells. IL-10 secretion was increased after *T. gondii* infection and was further increased in the infected Tim-3-neutralized group (Figure 5C). However, the TNF-α/IL-10 ratios were higher in infected cells than in uninfected cells and were further increased in infected Tim-3-neutralized cells (Figure 5D). *In vivo*, as the expression of Tim-3 decreased, TNF-α expression increased significantly in *T. gondii* infected mice; and the levels of increase was much higher in Tim-3^−/−^ infected mice. On the other hand, IL-10 levels were reduced by *T. gondii* infection, but increased significantly in Tim-3^−/−^ infected mice (Figure 5E). TNF-α/IL-10 ratios were higher in infected mice than in uninfected cells and were further increased in Tim-3^−/−^ infected mice (Figure 5F).

### Decrease of Tim-3 by *T.gondii* Infection Led to the Inhibition of PI3K-AKT Phosphorylation, Which Mediates the Transcription Factors C/EBPβ and SOCS1

**Figure 6:**
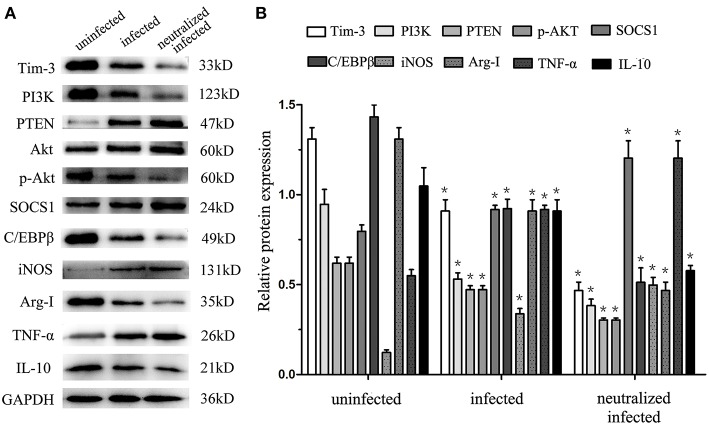
Downregulation of Tim-3 on human decidual macrophages leads to expression changes of signaling pathway molecules and macrophages functional indicators. Western blot analysis of Tim-3, PI3K, PTEN, AKT, pAKT, SOCS1, C/EBPβ, iNOS, Arg-I, TNF-α, IL-10, and GAPDH, which performed in uninfected, infected, and Tim-3-neutralized infected groups **(A,B)**. Differences were identified by unpaired *t*-test; *p < 0.05.

To obtain more details in the mechanisms of Tim-3-directed decidual macrophage polarization, western blotting was used to analyze the possibility that Tim-3 regulates PTEN/PI3K-AKT signaling, which, in turn, mediates the constitutively regulated promotors, C/EBPβ and SOCS1 of enzymes and cytokines in decidual macrophage. The results show that as the expression of Tim-3 decrease, the expression level of PI3K and phosphorylated AKT (pAKT) were decreased after *T. gondii* infection (Figures 6A,B). There was a more pronounced reduction of PI3K and pAKT in infected Tim-3-neutralized human macrophages (Figures 6A,B). We also examined PTEN expression, which antagonizes PI3K and reduces the AKT activation (21). With the decrease in Tim-3 levels due to *T. gondii* infection, PTEN expression increased and increased even further in the infected Tim-3-neutralized cells (Figures 6A,B). We also tested the expression of two crucial transcription factors, C/EBPβ and SOCS1, which are constitutively regulated promotors of macrophage functions related to enzymes and cytokines in human decidual macrophages. C/EBPβ expression decreased after *T. gondii* infection and further decreased in infected Tim-3-neutralized human decidual macrophages (Figures 6A,B). Analysis of SOCS1 showed that its expression was up-regulated following *T. gondii* infection and was further up-regulated in the infected Tim-3-neutralized human decidual macrophages (Figures 6A,B). Data also showed that along with decreased Tim-3, M1 functional indicators iNOS, and TNF-α expression were decreased, while M2 functional indicators Arg-I and IL-10 expression were elevated.

## Discussion

The immune micro-environment at the maternal-fetal interface plays an important role in implantation and embryonic and placental development (22, 23). *T. gondii* infection is known to lead to abnormal pregnancies by disrupting the delicate immunity and tolerance balance at the maternal-fetal interface (24, 25). Previously, we showed that functional molecules and cytokines produced by maternal NK cells, DC, and Tregs are affected by *T. gondii* infection, which then contributes to abnormal pregnancy (26–28). We also recently observed an important immune-regulatory role of an immune inhibitory molecule, LILRB4, in inducing the functional polarization of decidual macrophages, thus generating abnormal pregnancy outcomes due to *T. gondii* (13). Tim-3, a newly defined immune inhibitory receptor, down-regulates Th1 responses and is involved in tolerance induction (29). Tim-3 is also constitutively expressed on macrophages and it regulates the innate immune response by altering the function of macrophages (14). Nevertheless, whether Tim-3 participate in abnormal pregnancy outcomes during *T. gondii* infection and what its precise role is have been unclear.

To evaluate the effect of Tim-3 on abnormal pregnancy outcomes with *T. gondii*, Tim-3^−/−^ infected pregnant mouse models were established and their pregnancy outcomes were compared to those of *T. gondii* infected WT pregnant mice. Interestingly, pregnancy outcomes in Tim-3^−/−^ infected pregnant mice were more severe. This results strongly suggests the intimate involvement of Tim-3 in adverse pregnancy outcomes caused by *T. gondii* infection. Our results further show result showed that Tim-3 expression was significantly down-regulated on both human and mouse decidual macrophages after *T. gondii* infection. To determine the specific role of Tim-3 on the function of decidual macrophages, the expression levels of decidual macrophages functional molecules, including membrane molecules, arginine metabolic enzymes, and cytokines, were analyzed.

CD80 and CD86 are two important co-stimulatory molecules that reflect the activation status of the macrophages and induce T helper cell differentiation by their levels of expression (30, 31). M1 decidual macrophages are characterized by the high expression levels of CD80 and CD86, which are associated with inflammatory responses and poor maternal-fetal tolerance (7). Excessive expression levels of CD80 and CD86 reportedly give rise to a shift to a Th1 response at the maternal-fetal interface, thus contributing to the occurrence of miscarriage (11). Human decidual macrophages have properties predominantly associated with homeostatic M2 macrophages, including the expression of the homeostatic scavenger receptor CD163 and the pattern recognition receptors CD206 and CD209 (5). M2 cells have immunosuppressive capacities, contributing to tissue remodeling, and promoting Th2 response and maternal-fetal tolerance (32). Our previous study has demonstrated that *T. gondii* infection can affect M1- and M2- related membrane functional molecules of mouse decidual macrophages (13). In the present study, the results show that the any decrease in Tim-3 expression by *T. gondii* infection is associated with the up-regulation of M1 membrane functional molecules (CD80, CD86) and the down-regulation of M2 membrane functional molecules (CD206, CD163, CD209) both *in vitro* and *in vivo*.

To further explore whether changes in membrane functional molecules of decidual macrophages during *T. gondii* infection are due to decreased Tim-3 expression, infected human decidual macrophages with Tim-3-neutralized antibody and Tim-3^−/−^ infected pregnancy mouse model were established *in vitro* and *in vivo*. The data shows that in infected human decidual macrophages with Tim-3-neutralized antibody and decidual macrophages of Tim-3^−/−^ infected mice, M1 membrane-functional molecules (CD80, CD86) were more up-regulated, while M2 membrane-functional molecules (CD206, CD209, CD163) were more down-regulated than in infected human decidual macrophages and infected WT mice. These results suggest that the changing expression of membrane functional molecules from the M2 type to the M1 type is due to the down-regulation of Tim-3 expression by *T. gondii* infection. This means that Tim-3 down-regulation due to *T. gondii* infection promotes M1 decidual macrophage polarization and contributes to the abnormal pregnancy outcomes.

At the most fundamental level, M1/M2 polarity arises from arginine metabolism via two enzymatic pathways: inducible nitric oxide synthase (iNOS) and arginase Arg-I, which are functionally distinct and antagonistic (33). At the maternal-fetal interface, Arg-I is involved in immunosuppression and reportedly promotes polyamine synthesis to enhance placental growth and development (34). Conversely, iNOS has not been detected in normal pregnancy, and its excessive production may give rise to early embryo loss (35, 36). Arg-I and iNOS are increasingly being considered as indicators that differentiate between M2 and M1 decidual macrophages, respectively. In the current study, uninfected human or mouse decidual macrophages expressed little iNOS, which was induced after *T. gondii* infection. In contrast, Arg-I synthesis was significantly down-regulated in *T. gondii*-infected decidual macrophages both *in vitro* and *in vivo*. Therefore, *T. gondii* infection induces the expressions of iNOS and Arg-I to simultaneously enhance M1 and impair M2 decidual macrophages activation. *T. gondii* infected anti-Tim-3 neutralized human macrophages and Tim-3^−/−^ mice were used to determine whether changes in the arginine metabolic enzymes iNOS and Arg-I were due to decreased Tim-3 expression. Following Tim-3 blockage and *T. gondii* infection, iNOS expression became more enhanced while Arg-I synthesis became more reduced in decidual macrophages than those of infected decidual macrophages. These results suggest that the down-regulation of Tim-3 by *T. gondii* infection shifts M2 toward M1 decidual macrophage by dysregulating the arginine metabolic enzymes iNOS and Arg-I, which result in abnormal pregnancy outcomes.

IL-10 is constitutively produced by a variety of cells to counteract pro-inflammatory cytokines, which is considered an important homeostatic mechanism to avoid inappropriate T cell activation (37). In the context of normal pregnancy, decidual macrophages show an M2-polarized cytokine secretion pattern with abundant production of IL-10, which plays a vital role in the maternal immune tolerance of an allogeneic fetus (6, 38). Similarly, TNF-α is considered an important indicator of M1 decidual macrophages, and its increased secretion at the maternal-fetal interface leads to severe abnormal pregnancy outcomes (39, 40). Thus, the major cytokines TNF-α and IL-10 are secreted by decidual macrophages at the maternal-fetal interface and are involved in the balance of M1 and M2 phenotypes (5, 12). Studies have confirmed that the production of TNF-α and IL-10 by decidual macrophages change in a *T. gondii* infected state. TNF-α levels and TNF-α/IL-10 ratios were both increased after *T. gondii* infection *in vitro* and *in vivo*. Experiments were performed to further explore whether changes in TNF-α and IL-10 secretion during *T. gondii* infection were due to a decrease in Tim-3 expression. Flow cytometry and ELISA were used to analyze the expression of TNF-α and IL-10 expression by anti-Tim-3 human and Tim-3^−/−^ infected mouse decidual macrophages. Results show that TNF-α levels and TNF-α/IL-10 ratios were increased compared to the infected group. These results provide further evidence that Tim-3 down-regulation on decidual macrophages by *T. gondii* infection results in the previously observed changes in TNF-α and IL-10 expression, which is associated with the prevalence of M1 and impairment of M2 decidual macrophages. Furthermore, this imbalance may contribute to the development of abnormal pregnancy outcomes during *T. gondii* infection.

The above results reinforce the view that Tim-3 down-regulation by *T. gondii* infection results in M1 decidual macrophages polarization; however, the mechanism involved still need further exploration. In the current study, several related signaling molecules, all of which have been shown to contribute to the expression of macrophage polarization-associated enzymes and cytokines, were monitored.

Tim-3 signaling may increase PI3K-AKT phosphorylation, which is critical in altering TNF-α and IL-10 productions in macrophages (19). PI3K activation has been considered an essential step toward M2 activation of macrophages (41). The PI3K regulator, a lipid phosphatase known as a phosphatase and tensin homolog (PTEN), also contributes to macrophage polarization by suppressing the levels of Arg-I and M2 polarization, which is mediated by the increased expression and activation of the transcription factors C/EBPβ, suggesting that the regulation of PTEN/PI3K-AKT signaling is a central node for controlling macrophage polarization (42, 43). In addition, another signaling cascade downstream of PI3K-AKT, SOCS1, is a critical mediator in sustaining enhanced PI3K activity, which drives M2 activation. This not only promotes the production of the immunoregulatory cytokine IL-10 and M2 macrophage polarization, but also decreases the level of proinflammatory indicators iNOS and TNF-α (44, 45). However, whether PI3K/AKT signaling plays a role in decidual macrophages polarization due to Tim-3 down-regulation by *T. gondii* infection is still unclear. Our data shows that along with decreased Tim-3, the degree of phosphorylation of AKT, expression of PI3K and activation of C/EBPβ were suppressed, whereas the expression of PTEN and activation of SOCS1 was enhanced after *T. gondii* infection. Also, M1 functional indicators iNOS and TNF-α expression were decreased, M2 functional indicators Arg-I and IL-10 expression were elevated along with decreased Tim-3. Our data demonstrates causal relations between decreased Tim-3 expression and downstream signaling molecules, which contribute to the dysregulation of decidual macrophages functional indicators-enzymes (iNOS, Arg-I) and cytokines (TNF-α, IL-10). Our findings will be of great value in understanding how Tim-3 signals controlling decidual macrophage polarization and contribute to the adverse pregnancy outcomes of *T. gondii* infection.

In summary, Tim-3 down-regulation by *T. gondii* infection affects PI3K/AKT signaling involved in arginine enzymes expression and cytokine secretion, which results in decidual macrophage polarization toward to M1 phenotype. The imbalances in membrane molecules, arginine enzymes and cytokines of decidual macrophages induced by decreased Tim-3 contribute to abnormal pregnancy outcomes caused by *T. gondii* infection.

## Data Availability

All datasets generated for this study are included in the manuscript and/or the supplementary files.

## Ethics Statement

For human subjects:

This study was carried out in accordance with the recommendations of Ethics Committee of Binzhou Medical University with written informed consent from all subjects. All subjects gave written informed consent in accordance with the Declaration of Helsinki. The protocol was approved by the Ethics Committee of Binzhou Medical University.

For animal subjects:

This study was carried out in accordance with the recommendations of Institutional and National Institutes of Health guidelines. The protocol was approved by the Ethics Committee and Institutional Animal Experimental Ethics Committee of Binzhou Medical University.

## Author Contributions

DZ, LR, MZ, and XH designed the experiments. XL, LC, and TL contributed to sample collection. DZ, HZ, MZ, CY, XS, and YJ analyzed the data. DZ, LR, MZ, and XH wrote the manuscript. DZ and XH edited the manuscript.

### Conflict of Interest Statement

The authors declare that the research was conducted in the absence of any commercial or financial relationships that could be construed as a potential conflict of interest.
